# Supplementary material for: The Systems Biology Research Tool: evolvable open-source software
Source: BMC Syst Biol. 2008 Jun 29;2:55. doi: 10.1186/1752-0509-2-55 (PMC2446383; doi:10.1186/1752-0509-2-55)
Supplement: Additional file 1 — SBRT Archive. An archive of the current version of the Systems Biology Research Tool. [file 1752-0509-2-55-S1.zip › sbrt-1.4.0/doc/developers_guide/api/sbrt/shell/text/chem/class-use/RxnNameFormat.html]

Uses of Interface sbrt.shell.text.chem.RxnNameFormat


|  |  |  |
| --- | --- | --- |
| **Overview** \| **Package** \| **Class** \| **Use** \| **Tree** \| **Deprecated** \| **Index** \| **Help** | |  |
| PREV   NEXT | **FRAMES**    **NO FRAMES**     **All Classes** |  |


---


## **Uses of Interface sbrt.shell.text.chem.RxnNameFormat**

| Packages that use RxnNameFormat | |
| --- | --- |
| **sbrt.shell.text.chem** | Provides classes and interfaces for defining formats for chemistry related objects. |
| **sbrt.shell.text.fba** | Provides classes and interfaces for defining formats for objects relevant to Flux Balance Analysis. |

Uses of RxnNameFormat in sbrt.shell.text.chem

| Classes in sbrt.shell.text.chem that implement RxnNameFormat | |
| --- | --- |
| `class` | `RxnNameFormatV1`             This class is a concrete implementation of `RxnNameFormat`. |

Uses of RxnNameFormat in sbrt.shell.text.fba

| Classes in sbrt.shell.text.fba that implement RxnNameFormat | |
| --- | --- |
| `class` | `RxnNameVerifier`             This class is used to ensure reaction names exist in a given `Fluxome`. |

---


|  |  |  |
| --- | --- | --- |
| **Overview** \| **Package** \| **Class** \| **Use** \| **Tree** \| **Deprecated** \| **Index** \| **Help** | |  |
| PREV   NEXT | **FRAMES**    **NO FRAMES**     **All Classes** |  |


---
